# Supplementary material for: Setting a Course for Preventing Hepatitis E in Low and Lower-Middle-Income Countries: A Systematic Review of Burden and Risk Factors
Source: Open Forum Infect Dis. 2021 Apr 13;8(6):ofab178. doi: 10.1093/ofid/ofab178 (PMC8186248; doi:10.1093/ofid/ofab178)
Supplement: ofab178_suppl_Supplementary_Materials [file ofab178_suppl_supplementary_materials.zip › Supplemental Table 1.docx]

Supplemental Table 1. Risk factors for hepatitis E virus (HEV) infection, disease, or death. Household water sources and type of sanitation facilities were categorized as improved or unimproved according to 2019 World Health Organization/United Nations Children’s Fund standard categories for improved/unimproved water and sanitation

| **Risk Factor Type** | **Exposures hypothesized to increase the likelihood of HEV infection, disease, or death** |
| --- | --- |
| Water | -Unimproved household water sources  -Consumption of water from unimproved sources during a predetermined time frame  -Storage of water in a reservoir or wide mouthed container |
| Sanitation | -Unimproved household sanitation facility |
| Hygiene | -Poor hygiene behaviors |
| Animal related | -Contact with animals  -Occupations with possible contact with animals (e.g. farming)  -Presence of animals in the home or on one's property  -Use of dung (e.g. home construction, fuel) |
| Blood | -Receipt of blood transfusion, injections, or intravenous drugs  -Reporting cuts with blood to blood contact  -Touching other people's blood  -Shaving in a barbershop |
| Food and drink (non-water) | -Consumption of a specified food or non-water drink during a predetermined time frame |
| Age | -Older age |
| Sex | -Male sex/gender |
| Education | -Lower education |
| Household composition | -More people in the household  -More children in the household  -Higher ratio of household occupants to sleeping rooms in household |
| Other | -Lower household income and/or expenditures  -Lower quality (e.g. expense, durability) household construction materials  -Married  -Rural residence  -Recent travel to a town or city  -Rainy season  -Length of time spent at a farm  -Eating from a common bowl  -Contact with a jaundiced case  -Family history of hepatitis or jaundice  -Sexual contact  -Coinfections and comorbidities  -PROGINS gene mutation  -Pregnancy |
