# Supplementary figures and images for: Setting a Course for Preventing Hepatitis E in Low and Lower-Middle-Income Countries: A Systematic Review of Burden and Risk Factors
Source: Open Forum Infect Dis. 2021 Apr 13;8(6):ofab178. doi: 10.1093/ofid/ofab178 (PMC8186248; doi:10.1093/ofid/ofab178)

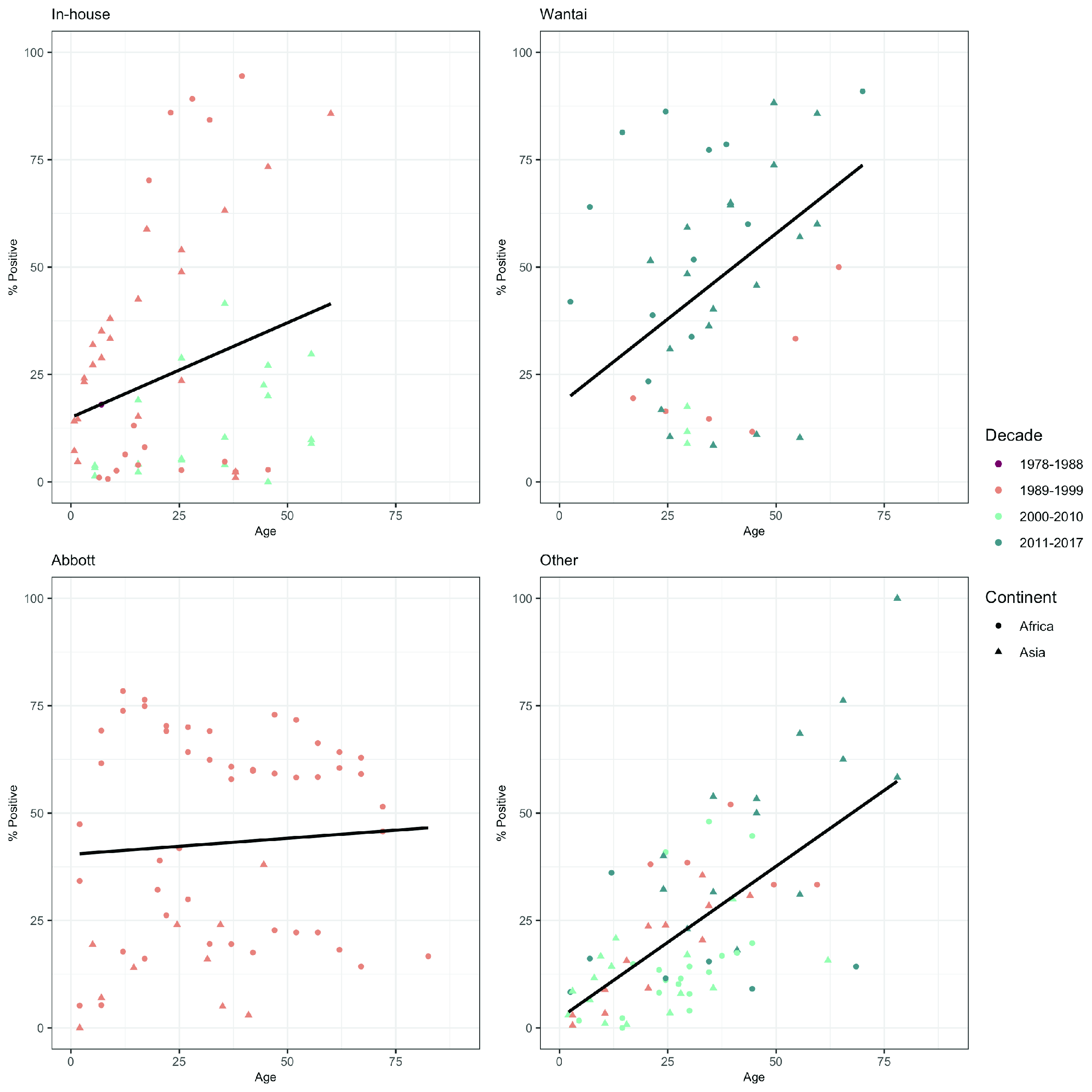

Supplement: ofab178_suppl_Supplementary_Materials [file ofab178_suppl_supplementary_materials.zip › Supplemental Figure 1.tif]
